# BUB1 drives the occurrence and development of bladder cancer by mediating the STAT3 signaling pathway

**DOI:** 10.1186/s13046-021-02179-z

**Published:** 2021-12-01

**Authors:** Ning Jiang, Yihao Liao, Miaomiao Wang, Youzhi Wang, Keke Wang, Jianing Guo, Peikang Wu, Boqiang Zhong, Tao Guo, Changli Wu

**Affiliations:** 1 Tianjin Institute of Urology, The Second Hospital of Tianjin Medical University, Tianjin, 300211 China; 2 Department of Pathology, The Second Hospital of Tianjin Medical University, Tianjin, 300211 China; 3 Sino-Singapore Eco-City Hospital of Tianjin Medical University, Tianjin, 300450 China

**Keywords:** Bladder cancer, BUB1, STAT3, Phosphorylation, Proliferation

## Abstract

**Background:**

The incidence of bladder urothelial carcinoma (UC), a common malignancy of the urinary tract, is approximately three times higher in men than in women. High expression of the mitotic kinase BUB1 is associated with the occurrence and development of several cancers, although the relationship between BUB1 and bladder tumorigenesis remains unclear.

**Methods:**

Using a microarray approach, we found increased BUB1 expression in human BCa. The association between BUB1 and STAT3 phosphorylation was determined through molecular and cell biological methods. We evaluated the impact of pharmacologic inhibition of BUB1 kinase activity on proliferation and BCa progression in vitro and in vivo.

**Results:**

In this study, we found that BUB1 expression was increased in human bladder cancer (BCa). We further identified through a series of molecular and cell biological approaches that BUB1 interacted directly with STAT3 and mediated the phosphorylation of STAT3 at Ser727. In addition, the findings that pharmacologic inhibition of BUB1 kinase activity significantly suppressed BCa cell proliferation and the progression of bladder cancer in vitro and in vivo were further verified. Finally, we found that the BUB1/STAT3 complex promoted the transcription of STAT3 target genes and that depletion of BUB1 and mutation of the BUB1 kinase domain abrogated this transcriptional activity, further highlighting the critical role of kinase activity in the activation of STAT3 target genes. A pharmacological inhibitor of BUB1 (2OH-BNPP1) was able to significantly inhibit the growth of BCa cell xenografts.

**Conclusion:**

This study showed that the BUB1 kinase drives the progression and proliferation of BCa by regulating the transcriptional activation of STAT3 signaling and may be an attractive candidate for therapeutic targeting in BCa.

**Supplementary Information:**

The online version contains supplementary material available at 10.1186/s13046-021-02179-z.

## Background

Bladder cancer (BCa) is the 8th leading cause of new cancer cases in men worldwide, and 81,400 new cases and 17,980 deaths occurred in the United States in 2020, according to statistical data [1]. At present, the main challenge in BCa treatment is the extremely high recurrence rate after cystectomy or adjuvant chemotherapy [2]. Thus, efforts to gain an in-depth understanding of the mechanisms of bladder carcinogenesis are strongly recommended. Cancer cells are characterized by uncontrolled proliferation and aneuploidy [3]. BUB1, a mitotic serine/threonine kinase, has multiple functions in chromosome segregation, KT-MT interaction and SAC function [4–6]. It has been found that BUB1 phosphorylates Cdc20 and histone H2A, resulting in active transcription in human cells [7, 8]. BUB1 was also found to be deregulated and linked to tumorigenesis, and mice with BUB1 mutations were found to exhibit increased chromosome segregation errors and aneuploidy [9–11]. Moreover, BUB1 overexpression was found to drive spontaneous tumorigenesis and accelerate Myc-induced lymphoma development in transgenic mice [12]. It was also found that overexpression of BUB1 was tightly associated with the development and progression of several human cancers, including breast cancer, prostate cancer, and hepatocellular carcinomas, as well as lymphoma and ovarian cancer [13–17]. Additionally, Aurora-B hyperactivation by BUB1 overexpression was found to result in chromosome missegregation, which induced tumor formation [13, 14]. The expression of BUB1 was found to be upregulated in basal-like breast cancer [17], and BUB1 was required for maintaining cancer stem cell renewal in breast cancer cell lines [18, 19]. STAT3 activation is frequently observed in malignant transformation to carcinoma, occurring in almost 45% of human BCa cases [20, 21]. Previous research found that treatment of Stat3 transgenic mice with BBN can induce rapid progression of BCa [22]. As a transcription factor, STAT3 is usually activated by cytokines or phosphorylation [23], and activated STAT3 regulates a series of transcriptional processes of critical target genes, such as POU5F1 and NFATC2, which are involved in cell survival, proliferation, differentiation and apoptosis [24, 25]. Although many previous studies have indicated that STAT3 plays a critical role in BCa development [22–26], mechanistic insights into how BUB1 mediates STAT3 to promote BCa largely remain to be elucidated.

In this study, we demonstrated the role of BUB1 as a novel, positive regulator of STAT3 activity. BUB1 interacted with STAT3 and phosphorylated STAT3 at Ser727. Furthermore, BUB1 was recruited to phosphorylate STAT3 and interacted with the transcriptional coactivator p300 to form a complex, which facilitated the enhancement of serine phosphorylation-dependent STAT3 transcriptional activity. Finally, BUB1 was found to enhance NFATC2- and LHX1-induced proliferation of BCa cells mediated by STAT3. Treatment with a pharmacological inhibitor of BUB1 (2OH-BNPP1) inhibited the growth of BCa cell xenografts. The BUB1 kinase may be an attractive candidate for therapeutic targeting in BCa.

## Materials and methods

### Patient samples

In this study, 34 BCa and matched paracancerous tissue samples were collected from BCa patients who underwent surgical resection without neoadjuvant chemotherapy or radiotherapy in the Department of Urology of the Second Affiliated Hospital of Tianjin Medical University (China) between 2017 and 2019. Detailed patient information is presented in Supplementary Table 1. The collection process of bladder cancer tissue was based on the specific criteria, the inclusion criteria: these patients underwent total cystectomy; the exclusion criteria: these patients underwent electrocystectomy and puncture. The processes used to obtain samples followed strict scientific methods and were approved by the Ethics Committee of the Second Hospital of Tianjin Medical University.

### Cell culture

T24, 5637 and EJ bladder cancer cells (ATCC; America) were cultivated in RPMI-1640 medium (Invitrogen) supplemented with 10% fetal bovine serum and antibiotics. RNAi oligonucleotides targeting BUB1 were purchased from GenePharma.

### Transfection and drugs treatment

**s**hBUB1 Wt (full-length BUB1 was cloned into pcDNA3.1) was donated by Dr. Gang-Zhang from Denmark, and the myc-BUB1 and myc-KD vectors were donated by Sabine Elowe from Canada. The S727A plasmid is from Addgene (#33102). We transfected bladder cancer T24, 5637 and EJ cells with 20uM siRNA, 2μg BUB1 related plasmid for subsequent invitro experiments. We also treated bladder cancer T24, 5637 and EJ cells with 1uM-50uM BUB1 inhibitor 2OH-BNPP1 for detecting IC50 value, and further treated these cells with 5uM/10uM 2OH-BNPP1 for subsequent invitro experiments.

### In vitro kinase assay

For the in vitro kinase assay, BUB1 or STAT3 was immunoprecipitated using the Flag or MYC epitope in NP-40 lysis buffer. Three hundred nanograms of purified BUB1 and 1 mg of STAT3/H2A were incubated in the presence or absence of the BUB1 inhibitor in kinase assay buffer containing 50 mM HEPES (pH 7.5), 15 mM MgCl_2_, 1 mM EGTA, 10% glycerol, 10 mM DTT and 0.1 mM ATP at 30 °C. After 30 min, the reaction product was separated by SDS–PAGE, and immunoblotting was then conducted with anti-STAT3-pSer727, anti-H2A-pT120, anti-total STAT3, anti-H2A, and anti-BUB1 antibodies.

### Co-Immunoprecipitation and Western blotting

T24, 5637 and EJ cells were harvested and lysed in lysis buffer (150 mM KCl, 75 mM HEPES (pH 7.5), 1.5 mM EGTA, 1.5 mM MgCl_2_, 10% glycerol, and 0.075% NP-40 supplemented with protease inhibitor cocktail [Roche, USA]). The lysate was precleared using a mixture of protein A–Sepharose (CL-4B; GE Healthcare) and the appropriate antibody overnight at 4 °C. Immunoprecipitates were washed with lysis buffer, resuspended in sample buffer, boiled and analyzed by SDS–PAGE. Individual samples (40 μg of protein) were separated on an 8% SDS polyacrylamide gel and transferred to PVDF membranes (Millipore, Billerica, MA). Membranes were blocked in a PBS-Tween 20 solution with 5% fat-free milk for 1 h at room temperature and were then incubated with appropriate dilutions of primary antibodies specific for STAT3 overnight at 4 °C. After washing, membranes were incubated with HRP-conjugated anti-rabbit or anti-mouse IgG for 1 h. Membranes were developed in an ECL mixture (Vector Laboratories, Burlingame, CA) and visualized by Imager.

### RNA extraction and analysis

RNA was extracted using TRIzol reagent (Invitrogen) according to the manufacturer’s instructions. RNA (2μg) was subjected to reverse transcription using Superscript III transcriptase (Invitrogen). The obtained cDNA was used for qPCR using a Bio–Rad CFX96 system with SYBR Green. Primers sequences are presented in Supplementary Table 2. RNA expression levels were normalized to that of GAPDH RNA.

### Luciferase assay

For the dual luciferase assay, 5637 cells were plated in triplicate into 12-well plates and cotransfected with 1 μg of the reporter construct, 15 pmol of the STAT3 luciferase reporter vector, and BUB1 siRNA by using transfection reagent (Roche). Transfected cells were cultured, and 24 h later, supernatants were collected for the luciferase assay using a Dual Luminescence Assay Kit (GeneCopoeia, MD) according to the manufacturer’s instructions. The luciferase assay was performed with the Glo Luciferase Assay System (Promega).

### Chromatin Immunoprecipitation

Five thousand six hundred thirty-seven cells were grown in RPMI-1640 medium (Invitrogen) supplemented with 10% charcoal-stripped FBS (CSF, HyClone, USA). Then, 1% formaldehyde was added for DNA crosslinking at room temperature for 10 min, and glycine was added (0.125 M final concentration) for 5 min to stop the crosslinking reaction. Cells were lysed with lysis buffer containing protease inhibitors, and DNA was then sonicated into fragments with a length between 200 and 1000 bp. One-tenth of the cell lysate was used as an input control, and the rest was used for immunoprecipitation with an anti-STAT3 antibody. After immunoprecipitation using protein G-agarose columns, protein-DNA complexes were eluted and heated at 65 °C to reverse crosslinking. After digestion with proteinase K, DNA fragments were purified using spin columns and analyzed using qPCR with the following thermal cycling parameters: 40 cycles at 94 °C for 30 s, 56 °C for 30 s, and 72 °C for 1 min. Specific primer sets were designed to amplify target sequences within the human NFATC2 and LHX1 promoters, as shown in Supplementary Table 2.

### RNA-Seq analysis

A total of 5637 cells transfected with BUB1 or scramble siRNA were used for RNA-seq. RNA quantity and quality were evaluated by an Agilent 2100 Bioanalyzer (Agilent Technologies, Santa Clara, CA, USA). Four micrograms of total RNA was used to generate sequencing libraries, and a KAPA Stranded RNA-Seq Library Prep Kit from Illumina (San Diego, CA, USA) was used according to the manufacturer’s instructions. First, mRNA enrichment was performed with the NEBNext® Poly (A) mRNA Magnetic Isolation Module. After heating, RNA was fragmented into small pieces using divalent cations for purification. The cleaved RNA fragments were reverse transcribed into first-strand cDNA using reverse transcriptase and random primers, and second-strand cDNA synthesis was then performed using DNA polymerase I and RNase H. The products were purified and enriched by polymerase chain reaction (PCR) to generate the final cDNA library. The enrichment and size distribution of the library were tested with an Agilent 2100 Bioanalyzer. Hybridization and cluster generation were performed in a cBot system. Samples were subjected to single-end sequencing with a read length of 50 bp in an Illumina HiSeq 4000 sequencer. After preprocessing of the raw reads (filtering and QC), the sequencing reads were mapped to the human reference genome version 19 using the TopHat algorithm version 2.0.9 with Ensembl gene annotations version GRCh37.65. Further analysis was performed with the R statistical programming language version 2.15.0. The DEGseq package was used to compare differentially expressed transcripts among samples based on FPKM values calculated by Ballgown (fold change≥2.0, *P*value ≤ 0.05). These genes positively regulated by BUB1 signaling were considered to be those with downregulated expression after BUB1 knockdown (BUB1 knockdown versus siSCR) and were subjected to KEGG pathway analysis. The *P* values for each gene in the two signaling pathways are listed in Supplementary Table 3. The accession number for the RNA-seq data is GSE130145.

### MTT assay

We transfected T24 and 5637 cells with si BUB1 for 48 h or treated T24 and 5637 cells with 0, 5 and 10 μM 2OH-BNPP1 (a BUB1 inhibitor) for 4 h. A total of 2.0 × 10^3^ cells per well were seeded into 96-well plates and cultured at 37 °C for 24 h, 48 h, 72 h, and 96 h. Then, 30 μL of MTT solution was added to each well at the indicated time, and the cells were cultured for another 2 h at 37 °C. The MTT solution was then removed, and 150 μL of dimethyl sulfoxide (DMSO) was added to each well. Finally, we measured the absorbance at 490 nm with a microplate reader.

### Transwell invasion assay

We transfected T24 and 5637 cells with si BUB1, BUB1-wt and BUB1 K821R plasmids, separately, or treated T24 and 5637 cells with 10 μM 2OH-BNPP1 for 4 h. In addition, 2 × 10^4^ cells were plated into the top chambers of 24-well Transwell plates, and 800 μl of RPMI-1640 medium containing 10% FBS was added to the bottom chambers. After an additional 48 h of incubation at 37 °C, we washed the chamber inserts twice with PBS, further fixed them with paraformaldehyde and stained them with crystal violet. Then, the number of invading cells in all chambers was calculated in 5 fields of view.

### Immunohistochemical staining

All clinical patient tissues were collected from the Department of Urology of the Second Hospital of Tianjin Medical University and were further preserved in formalin. In addition, mouse bladders and nude mouse subcutaneous tumors were removed in a sterile environment and were further preserved in formalin. All of the above tissues were frozen, embedded in paraffin and cut into 4 μm sections. These sections were handled and subjected to immunohistochemical staining according to the protocol. The corresponding antibodies were as follows: anti-BUB1 (DF6698, Affinity, 1:100); anti-ki67 (AF0198, Affinity, 1:100); anti-LHX1 (DF4823, Affinity, 1:100); and anti-NFATC2 (ab169140, Abcam, 1:100). The expression levels of BUB1, ki67, LHX1 and NFATC2 were evaluated and images acquired under a Zeiss microscope (200×).

### Immunofluorescence analysis

Cells were grown on Labtek II-CC2 chamber slides (Nunc), fixed with 4% paraformaldehyde for 5 min, washed with PBS and blocking buffer (PBS with 5% BSA and 0.1% Triton X-100), and incubated overnight at 4 °C with primary antibodies against BUB1 and STAT3. Alexa Fluor 350-, 488- or 647-conjugated donkey anti-mouse, anti-rabbit or anti-goat secondary antibodies (Invitrogen) were used. Cell nuclei were visualized with DAPI (Sigma).

### Sphere formation assay

Single-cell suspensions (1 × 10^3^ cells in 60 μl of medium) were mixed with 60 μl of cold Matrigel, and the mixture was placed along the rim of 24-well plates. The culture plates were placed in a 37 °C incubator for 10 min for solidification of the mixture, and 500 μl of medium was then added into each well. Spheres were counted under an Olympus light microscope after 7–14 days, and size differences were examined. At least three experiments were performed.

### Immunofluorescence

All patient tissues were collected from our hospital and fixed with formalin, embedded in paraffin and sliced into 4 μm sections. These sections were handled and subjected to immunofluorescence analysis according to the protocol. The sections were processed through the following procedure: dewaxing, antigen retrieval with sodium citrate, blocking with BSA and incubation with the indicated primary antibody overnight. The next day, the sections were incubated with the secondary antibody and DAPI and were subsequently observed and photographed.

Cells were transfected with the indicated plasmid for 48 h, digested with trypsin and plated at 1 × 10^4^ cells per well in a 24-well plate containing cell climbing slides. The climbing slides were handled and subjected to immunofluorescence analysis according to the protocol, which included the following steps: fixation with 4% paraformaldehyde, incubation with 0.5% Triton X-100, blocking with 5% BSA and incubation with the primary antibody overnight. We then sequentially incubated these climbing slides with the secondary antibody and DAPI and further observed and photographed the slides.

### Animal studies

Eight-week-old male BALB/c mice (HFK Bio-Technology Co. Ltd., Beijing) were injected subcutaneously with 2 × 10^6^ 5637 cells suspended in 0.1 mL of Matrigel (BD Biosciences) in the bilateral dorsal flanks. Once the tumors reached approximately 5 mm in length, the mice were randomized and treated daily with 2OH-BNPP1 (100 mg/kg body weight) by intraperitoneal injection for 9 days. Tumors were measured with digital calipers, and volumes were estimated using the formula LW^2^/2, where L = tumor length and W = tumor width. At the end of the studies, the mice were killed, and the tumors were excised and weighed. All procedures involving mice were approved by the University Committee on the Use and Care of Animals at Tianjin Medical University and conformed to all regulatory standards. We also established a model of spontaneous bladder cancer model in KM mice via feeding water supplemented with 0.1% BNN (N-butyl-N-(4-hydroxybutyl) nitrosamine), which induces bladder cancer. These mice were fed BNN for 3 months and killed 3 mice per month, and the bladders were extracted for further H&E and IHC staining. Drug treatment was stopped in the remaining mice fed BNN for 3 months; and these mice were killed, and bladders were excised bladders each month. The excised bladders were embedded in paraffin and sectioned for subsequent IHC and H&E staining. H&E staining was performed to assess structural changes, while IHC staining was conducted to evaluate BUB1 expression in the corresponding mouse bladders.

### Statistical analysis

Data are expressed as the mean ± SD values. Differences between two samples were determined by Student’s t-test. *P* values of 0.05 or less were considered statistically significant. Tumor weights were analyzed using GraphPad Prism 8.

## Results

### BUB1 expression was increased in human bladder cancer

**Fig. 1:**
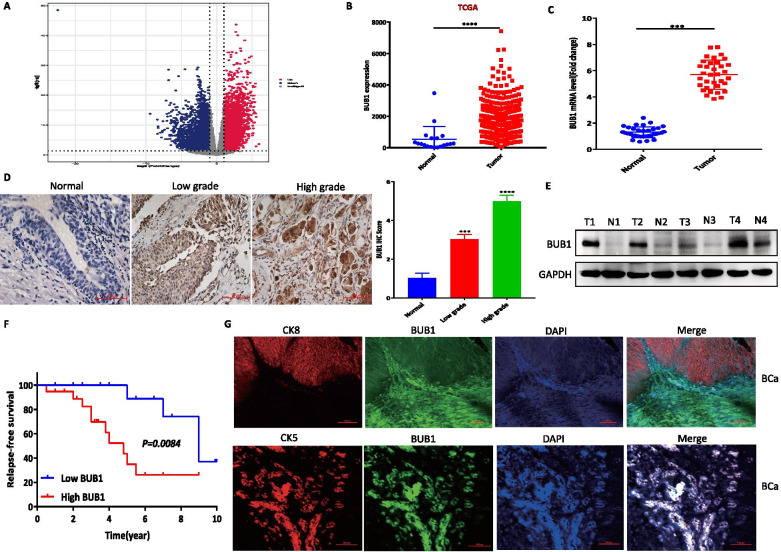
BUB1 expression was increased in human bladder cancer. **A**. Volcano plot of differentially expressed genes (DEGs) in TCGA BLCA data. **B**. Box plots of the relative mRNA expression levels of BUB1 in human bladder cancer samples from TCGA datasets. The boxes show that the expression level of BUB1(*n* = 19) in normal bladder tissue was lower than that (*n* = 411) in bladder cancer tissue. *P* < 0.0001. **C**. Real-time PCR analysis of BUB1 mRNA levels in tissue derived from 34 paired bladder cancer specimens. *P* < 0.001. **D**. Immunohistochemical (IHC) analysis of the BUB1 protein in human normal bladder and bladder cancer clinical samples. Quantification of IHC staining in human normal bladder and bladder cancer clinical samples. **E**. WB analysis of the BUB1 protein level in human normal bladder (N) and bladder cancer (T) clinical samples. **F**. Comparison of survival rates in the indicated groups. Low BUB1 expression group, *n* = 14; high BUB1 expression group, *n* = 20. Patients with high expression of BUB1 had a significantly worse prognosis than those with low expression of BUB1 (*P* = 0.0084); n: number of patients. **G**. Immunofluorescence staining showing the correlation between BUB1 localization and CK5 and CK8 localization in bladder cancer tissue

The functions of BUB1 during the progression and carcinogenesis of BCa are not fully understood. Comprehensive analysis of the expression profiles of 8739 genes in The Cancer Genome Atlas (TCGA) showed that BUB1 was upregulated in BCa tissue compared with normal bladder tissue (Fig. 1A, B). To further verify these TCGA data, we investigated the mRNA expression of BUB1 in 34 collected paired bladder cancer tissues, and the results showed that the mRNA expression of BUB1 was significantly higher in bladder cancer tissues than in normal tissues (Fig. 1C). Furthermore, we collected 34 paired bladder cancer tissues from our hospital and analyzed the expression of BUB1 in these patient samples by immunohistochemical staining. The results showed that BUB1 expression in bladder cancer was upregulated compared with that in normal bladder tissue. Interestingly, the expression of BUB1 in low-grade bladder cancer was lower than the expression of BUB1 in high-grade bladder cancer. We also found that the expression of BUB1 in the tumor epithelium progressively increased in BCa tissue compared with normal bladder tissue (Fig. 1D). We further divided these patients into high and low BUB1 expression groups and analyzed the correlation between BUB1 expression and several common clinicopathological features of bladder cancer (including age, sex, tumor grade, tumor size and metastasis status). We found that BUB1 was related to tumor size (X^2^ = 11.7588, *P* = 0.0006, Table 1) but not to age, sex, tumor grade or metastasis (*P* > 0.05). Moreover, we further found that the protein expression level of BUB1 in BCa was obviously higher compared with that in normal bladder tissue, consistent with the mRNA expression results (Fig. 1E; Supplementary Fig. 1). Based on the above results, we determined that BUB1 might be upregulated in BCa and promote the occurrence and progression of BCa. Importantly, survival analysis of patients stratified by BUB1 expression in two datasets was performed according to the Kaplan–Meier method. Differences in survival times were assessed using the log rank test. Higher expression levels of BUB1 were significantly associated with poorer overall survival (Fig. 1F). Previous studies showed that CK5 was mainly expressed in luminal cells, while CK8 was mainly expressed in basal cells. To further explore the location of BUB1 expression, we conducted immunofluorescence staining of CK5, CK8 and BUB1 in human bladder cancer tissue, and the results demonstrated that the location of BUB1 expression was consistent with the location of CK5 expression, while the location of CK8 expression was opposite that of BUB1 expression (Fig. 1G). And the occurrence of BCa was closely related to luminal cells, not basal cells. Collectively, the above results showed that BUB1 was mainly located in basal cells and was strongly associated with bladder carcinogenesis. The specific internal mechanism by which BUB1 regulates bladder cancer occurrence and development needs to be further studied.

**Table 1:** Relationships of BUB1 and clinicopathological characteristics in 34 patients with bladder cancer

Feature	All ***n*** = 34	BUB1 expression		χ^***2***^	***P***
		Low ***n*** = 14	High ***n*** = 20		
**Age (year)**				0.0038	0.9509
< 55	16	6	10		
≥ 55	18	8	10		
**Gender**				2.29E-31	1
Male	19	8	11		
Female	15	6	9		
**Tumor grade**				3.12 E-11	0.9862
Low	15	8	7		
High	19	6	13		
**Tumor size**				11.7588	0.0006*
< 3 cm	16	12	4		
≥ 3 cm	18	2	16		
**Metastasis**				0.1035	0.7477
Yes	12	4	8		
No	22	10	12		

### BUB1 was related to JAK-STAT signaling

**Fig. 2:**
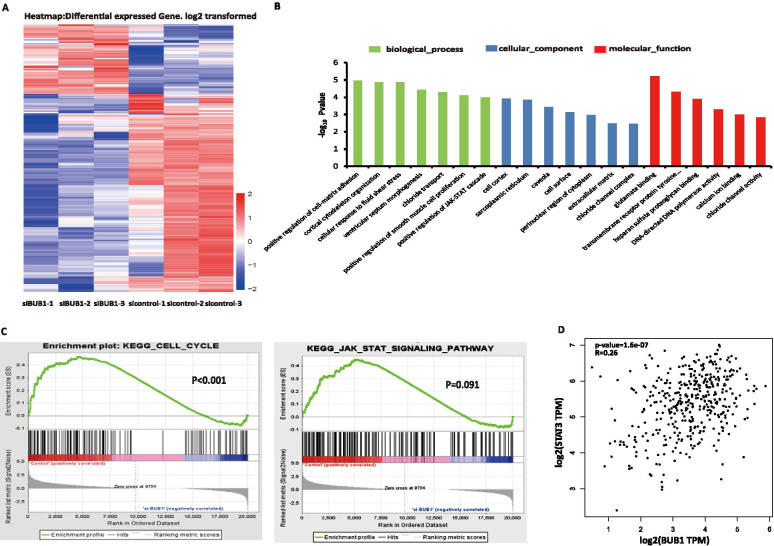
BUB1 was related to JAK-STAT signaling. **A**. Heatmap of RNA-seq expression data for 5637 cells transfected with si control or si BUB1. **B**. Gene Ontology annotations of 392 genes involved in biological processes, molecular function and cellular compartments. **C**. GSEA of RNA-seq data using gene sets from MSigDB revealed that BUB1 target genes were involved in cell cycle signaling and JAK-STAT signaling. **D**. The correlation between BUB1 and STAT3 based on GEPIA database analysis

To understand the biological significance of BUB1-mediated phosphorylation and the effects of BUB1 on the occurrence and progression of BCa, we investigated the transcriptomes of 5637 cells with BUB1 knockdown by high-throughput RNA deep sequencing (RNA-seq). In brief, total RNA was extracted from 5637 cells transfected with control siRNA or BUB1 siRNA (Fig. 2A; Supplementary Table 3). Gene Ontology (GO) analysis revealed that the enriched genes were mainly involved in the processes of cell adhesion and positive regulation of JAK-STAT signaling (Fig. 2B). Interestingly, downregulation of BUB1 resulted in the maximal influence on the expression of 1697 genes. The expression of 1246 genes was reduced in response to BUB1 knockdown, indicating that BUB1 positively regulated the expression of these genes. Conversely, 451 genes, including P53, PTEN and AR, were upregulated, indicating that BUB1 suppressed the expression of these genes (Fig. 2A). There were almost 3159 genes previously reported to be transcribed by STAT3, and we found that 392 of these target genes were co-targeted by both BUB1 and STAT3 (Supplementary Table 4; Supplementary Fig. 2 A-D). Further experiments showed that the expression of 183 of these cotargeted genes was reduced in response to BUB1-siRNA, indicating that BUB1 positively regulated the expression of these genes. Conversely, BUB1 suppressed the expression of 209 of the cotargeted genes, including BEST1. Using gene set enrichment analysis (GSEA), we observed a previously unknown and significant overlap of the BUB1-mediated transcriptome with JAK-STAT signaling and cell cycle signaling (Fig. 2C). We also found that BUB1 was positively related to STAT3 based on TCGA data in the online GEPIA database (R = 0.21; *P* < 0.05; Fig. 2D), which further supports the hypothesis that BUB1 might regulate the progression and prognosis of bladder cancer by mediating the STAT3 signaling pathway. Further experiments need to be performed to verify the role of BUB1/STAT3 in the occurrence and development of BCa.

### Molecular interaction between STAT3 and BUB1

**Fig. 3:**
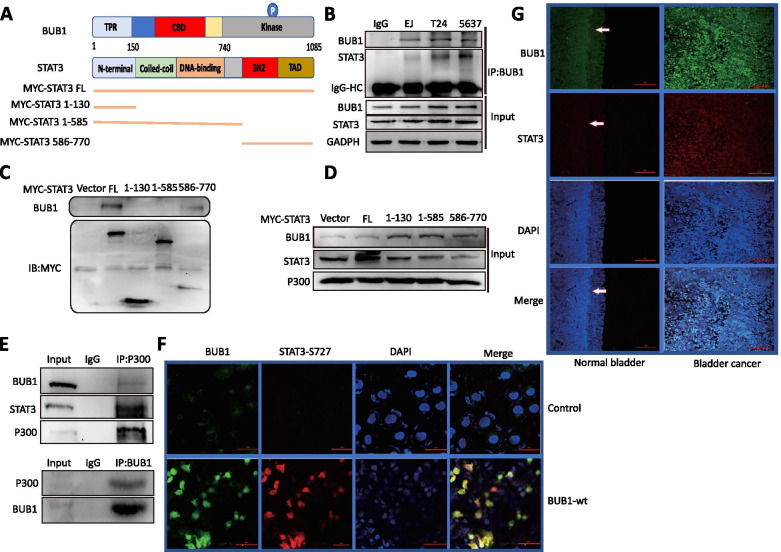
Molecular Interactions between STAT3 and BUB1. **A**. Schematic drawing of the domains and motifs of human BUB1 and STAT3. TPR: tetratricopeptide repeat. **B**. Co-IP and WB analysis of BUB1 and STAT3 proteins in total lysates obtained from T24, EJ and 5637 cells grown under serum-fed conditions. A mixture (1:1:1 ratio) of lysates from T24, EJ and 5637 cells was used in the IgG control sample. **C**. Co-IP and WB analysis of BUB1 and fragments of STAT3. **D**. Co-IP and WB analysis of STAT3 fragments and BUB1 in the input sample. **E**. Co-IP and WB analysis of the BUB1-P300 complex. **F**. Five thousand six hundred thirty-seven cells were transfected with the BUB1 wild-type vector for 24 h. Alexa Fluor 488 was used to stain BUB1 (green), Cy3 was used to stain STAT3 (red) and DAPI was used to stain cell nuclei (blue) for co-immunofluorescence (co-IF) analysis of the BUB1 and STAT3 proteins. **G**. Immunofluorescence staining (IF) of STAT3 (red) and BUB1 (green) in normal bladder and BCa tissues. Cell nuclei were visualized by DAPI staining

The BUB1 kinase protein contains multiple functional domains (Fig. 3A). Gang et al.’s SALIC proteomic data showed that BUB1 interacted with STAT [27]. To observe whether native BUB1 and STAT3 biochemically interact with each other in vitro, we performed coimmunoprecipitation (co-IP) and Western blot (WB) analysis with the EJ, T24, and 5637 BCa cell lines. We further constructed several fragments of STAT3 for subsequent experiments based on its functional domains (Fig. 3A). The results demonstrated that endogenous BUB1 interacted with STAT3 and formed a protein complex (Fig. 3B). The interactions between BUB1 and STAT3 were specific and especially strong in BCa 5637 cells. Next, to identify the regions of STAT3 involved in the BUB1-STAT3 interaction, various deletion mutants of myc-tagged STAT3 were constructed (Fig. 3A) and subjected to pulldown assays in 5637 cells. As shown in Fig. 3C, FL STAT3 and the C-terminal (amino acids 586–770) domain of STAT3 interacted moderately with BUB1. Several studies have shown that the histone acetyltransferase p300 binds STAT3 to form a complex and mediates STAT3 acetylation, resulting in a cooperative interaction promoting transcription [28]. Therefore, we investigated the interactions of STAT3 and p300 with BUB1 in 5637 cells by CO-IP. The results showed that STAT3 and p300 formed a stable complex with BUB1 (Fig. 3D, E). Moreover, coimmunofluorescence analysis demonstrated that compared with their localization in vehicle control cells, BUB1 and STAT3 proteins colocalized in the nuclei of 5637 cell with BUB1 overexpression (Fig. 3F). Taken together, these findings indicated that BUB1 interacted with STAT3 and activated the STAT3 protein and further translocates into the nucleus to regulate transcriptional processes in BCa cells. Furthermore, the results of immunofluorescence staining showed that the protein expression levels of BUB1 and STAT3 were significantly higher in BCa patient tissue than in normal bladder tissue and further revealed that endogenous BUB1 and STAT3 were more predominantly expressed in the nuclei of BCa cells than normal bladder cells, again confirming the relationship between elevation of BUB1 expression and activation of STAT3 in BCa cells (Fig. 3G).

### Knockdown of BUB1 attenuates the STAT3 transcriptional program

**Fig. 4:**
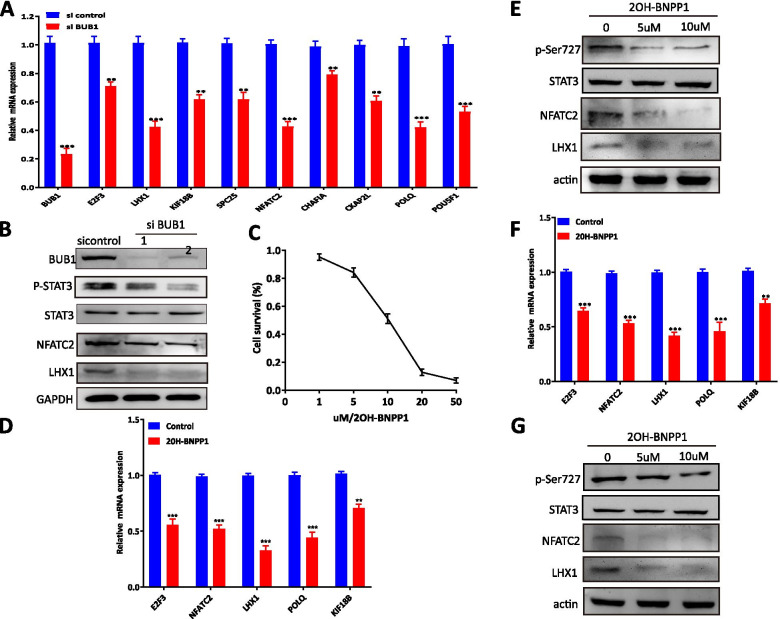
Knockdown of BUB1 Attenuates the STAT3 Transcriptional Program. **A**. 5637 cell were treated with siBUB1. qRT–PCR analysis was performed to measure the mRNA levels of STAT3–induced target genes. The data are presented as the means ± SDs. **B**. The protein levels of BUB1, Ser727-STAT3, STAT3, NFATC2, LHX1 and GAPDH were measured by Western blotting in siBUB1-transfected 5637 and T24 cells. **C**. MTT experiments showed the IC50 value of BUB1 inhibitor 2OH-BNPP1. **D**. 5637 cell were treated with 10 μM 2OH-BNPP1. qRT–PCR analysis was performed to measure the mRNA levels of STAT3–induced target genes. The data are presented as the means ± SDs. **E**. The protein levels of BUB1, Ser727-STAT3, STAT3, NFATC2, LHX1 and GAPDH were measured by Western blotting in 10 μM 2OH-BNPP1-treated 5637 cell. **F**. T24 cells were treated with 10 μM 2OH-BNPP1. qRT–PCR analysis was performed to measure the mRNA levels of STAT3–induced target genes. The data are presented as the means ± SDs. **G**. The protein levels of BUB1, Ser727-STAT3, STAT3, NFATC2, LHX1 and GAPDH were measured by Western blotting in 10 μM 2OH-BNPP1-treated T24 cells

To confirm this conclusion, we performed qRT–PCR by knocking down BUB1 with siRNA, and the results showed that the transcriptional activity of STAT3 was reduced significantly and that the expression of STAT3 target genes, including E2F3, KIF18B, LHX1, NFATC2, POLQ and so on, was diminished (Fig. 4A). The protein expression levels of the target genes LHX1 and NFATC2 were also obviously decreased in 5637 and T24 cells transfected with BUB1 siRNA (Fig. 4B). These results showed that BUB1 deletion suppressed the transcriptional activity of STAT3 and its corresponding transcriptional targets. To further confirm the role of BUB1 in the transcriptional activity of STAT3, we treated 5637 cells with a catalytic small molecule inhibitor of BUB1, 2OH-BNPP1, which was verified to be a specific inhibitor of BUB1 with IC_50_ values of approximately 10 μM (Fig. 4C). Furthermore, as an outcome of BUB1 kinase suppression by 2OH-BNPP1, a significant reduction in the mRNA and protein expression of the cotargeted genes LHX1 and NFATC2 was observed in 5637 and T24 cells treated with 2OH-BNPP1 (Fig. 4D-G), suggesting that BUB1 is required for maintaining the mRNA expression of the STAT3 target genes LHX1 and NFATC2. Collectively, the above results implied that BUB1 might promote the occurrence and development of bladder cancer by regulating the transcriptional activation of STAT3 signaling. In addition, targeting BUB1-STAT3 might be a potential feasible therapeutic strategy for many patients with bladder cancer.

### BUB1 kinase activity was required to maintain STAT3 target gene mRNA levels

**Fig. 5:**
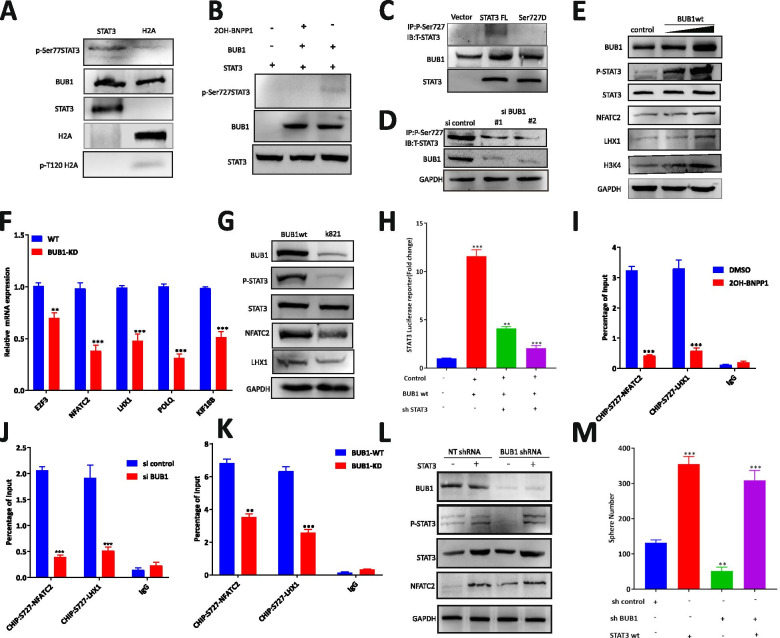
BUB1 Kinase Activity Was Required to Maintain STAT3 Target Gene mRNA Levels. **A**. STAT3 was purified alone or incubated with equimolar amounts of purified BUB1 in the absence or presence of 10 μM 2OH-BNPP1 and was then subjected to immunoblotting using the indicated antibodies. **B**. An in vitro kinase assay was performed using purified BUB1 and the indicated STAT3 protein, and immunoblotting was then performed with the indicated antibodies. **C** 5637 cell were cotransfected with BUB1 and MYC-tagged STAT3 or mutants, and immunoblotting was then performed. **D**. 5637 cell were transfected with si control or two distinct BUB1 siRNAs. Lysates were immunoprecipitated with the anti-pS727-STAT3 antibody, and immunoblotting was then performed with the anti-STAT3 antibody. Lysates were also subjected to immunoblotting with the anti-BUB1 antibody. **E**. The protein levels of BUB1, Ser727-STAT3, STAT3, NFATC2, LHX1 and GAPDH were measured by Western blotting in BUB1wt vector-transfected 5637 cell. **F**. 5637 cell were transfected with the BUB1wt vector or BUB1 KD vector. qRT–PCR analysis was performed to measure the mRNA levels of STAT3–induced target genes. The data are presented as the means ± SDs. **G**. The protein levels of BUB1, Ser727-STAT3, STAT3, NFATC2, LHX1 and GAPDH were measured by Western blotting in BUB1wt vector- or BUB1 KD vector-transfected 5637 cell. **H**. STAT3 luciferase activity in 5637 cell after transfection with BUB1wt and shSTAT3. The signal was quantified, and statistical significance was determined by Student’s t-test (**p < 0.01, ***p < 0.001). **I**. Chromatin prepared from 5637 cell treated with 2OH-BNPP1 was subjected to ChIP using an anti-STAT3-Ser727 antibody, and qPCR was then performed using primers targeting STAT3 or the control (gene desert) region. *p < 0.05, **p < 0.01. **J**. Chromatin prepared from 5637 cells treated with siBUB1 was subjected to ChIP using an anti-STAT3-Ser727 antibody. **K**. Chromatin prepared from 5637 cell treated with BUB1wt or KD vectors was subjected to ChIP using an anti-STAT3-Ser727 antibody. **L**. Western blot analysis of protein expression in 5637 cell after transfection with STAT3wt and shBUB1. Signals were quantified, and statistical significance was determined by Student’s t-test (**p < 0.01, ***p < 0.001). **M**. Sphere formation assay in STAT3wt vector- and shBUB1 vector-transfected 5637 cell

To further explore the relationship between BUB1 and STAT3 in BCa, we examined whether BUB1 regulates the activation of STAT3 by phosphorylation. First, an in vitro kinase assay was performed in 5637 cells. We incubated purified recombinant human BUB1 protein with purified STAT3 protein and H2A histone as a positive control, and we then performed immunoblotting with phospho-specific anti-STAT3-pSer727 or anti-H2A-T120 antibodies (Fig. 5A). As expected, the presence of the band representing phosphorylated STAT3 only after BUB1 was incubated with STAT3 indicated that BUB1 phosphorylated and activated STAT3 at Ser727; in addition, STAT3 Ser727 phosphorylation was abolished upon 2OH-BNPP1 treatment (Fig. 5B). To further validate the phosphorylation of STAT3 Ser727 by BUB1, a vector expressing the Ser727D point mutant was used for subsequent experiments. We observed that BUB1 phosphorylated STAT3 at Ser727 but failed to phosphorylate the Ser727D-STAT3 mutant (Fig. 5C). To evaluate the status of STAT3 phosphorylation depending on BUB1 activation, we used a small interfering RNA (siRNA) targeting BUB1. BUB1 knockdown resulted in a significant decrease in STAT3 Ser727 phosphorylation (Fig. 5D). Collectively, the above results suggested that the kinase activity of BUB1 was required for STAT3 phosphorylation. To further determine whether the kinase activity of the BUB1 protein facilitates the transcription of STAT3, 5637 cells were transfected with the BUB1 wt vector and BUB1 KD vector. Overexpression of BUB1 increased STAT3 phosphorylation and the expression of NFATC2 and LHX1 but did not change the expression of STAT3 in 5637 cells (Fig. 5E). The results of real-time PCR revealed that the mRNA expression of NFATC2, LHX1 and POLQ was significantly suppressed in KD vector-transfected BCa cells (Fig. 5F). In addition, a modest decrease in the mRNA level of E2F3 was observed in 5637 cells transfected with the KD vector. Notably, mutation of the BUB1 kinase domain (K821) did not increase STAT3 phosphorylation or the expression of NFATC2 and LHX1 (Fig. 5G). These data suggested that BUB1 KD can significantly suppress STAT3 transcription and the protein expression of the STAT3 targets NFATC2 and LHX1. To further determine the physiological relationship between BUB1 and STAT3, we analyzed the luciferase activity of STAT3 and found that overexpression of BUB1 increased STAT3 transcriptional activity, while treatment with shSTAT3 reversed this effect (Fig. 5H). Subsequent ChIP-qPCR revealed specific recruitment of STAT3 to the promoters of NFATC2 and LHX1, which was abolished after treatment with 2OH-BNPP1. These results suggested that the deposition of pS727-STAT3 was abolished by the BUB1 inhibitor (Fig. 5I; Supplementary Fig. 2 E, F). Furthermore, BUB1 knockdown by siRNA significantly reduced pS727-STAT3 deposition at the promoters of NFATC2 and LHX1 (Fig. 5J), consistent with the above results. In addition, BUB1 kinase KD significantly decreased the deposition of pS727-STAT3 at the NFATC2 and LHX1 promoters (Fig. 5K). Taken together, these results indicated that BUB1 phosphorylated STAT3 on Ser-727 and formed a complex at the promoters of STAT3 target genes. Collectively, the above results demonstrated that BUB1 controlled the expression of these target genes by phosphorylating STAT3 and further regulating transcriptional processes. To confirm STAT3 activation as the critical molecular mechanism by which BUB1 regulates the expression of STAT3 downstream genes, we further explored whether the expression of constitutively active STAT3 would rescue the expression of target proteins downregulated by BUB1 downregulation. The results showed that STAT3 rescued the phosphorylation of STAT3 S727 and NFATC2 (Fig. 5L). Interestingly, overexpression of STAT3-Flag in BCa cells not only restored their proliferation but also reversed the impairment of tumor sphere formation caused by BUB1 knockdown (Fig. 5M).

### BUB1 promoted the proliferation and invasion of bladder cancer T24 and 5637 cells

**Fig. 6:**
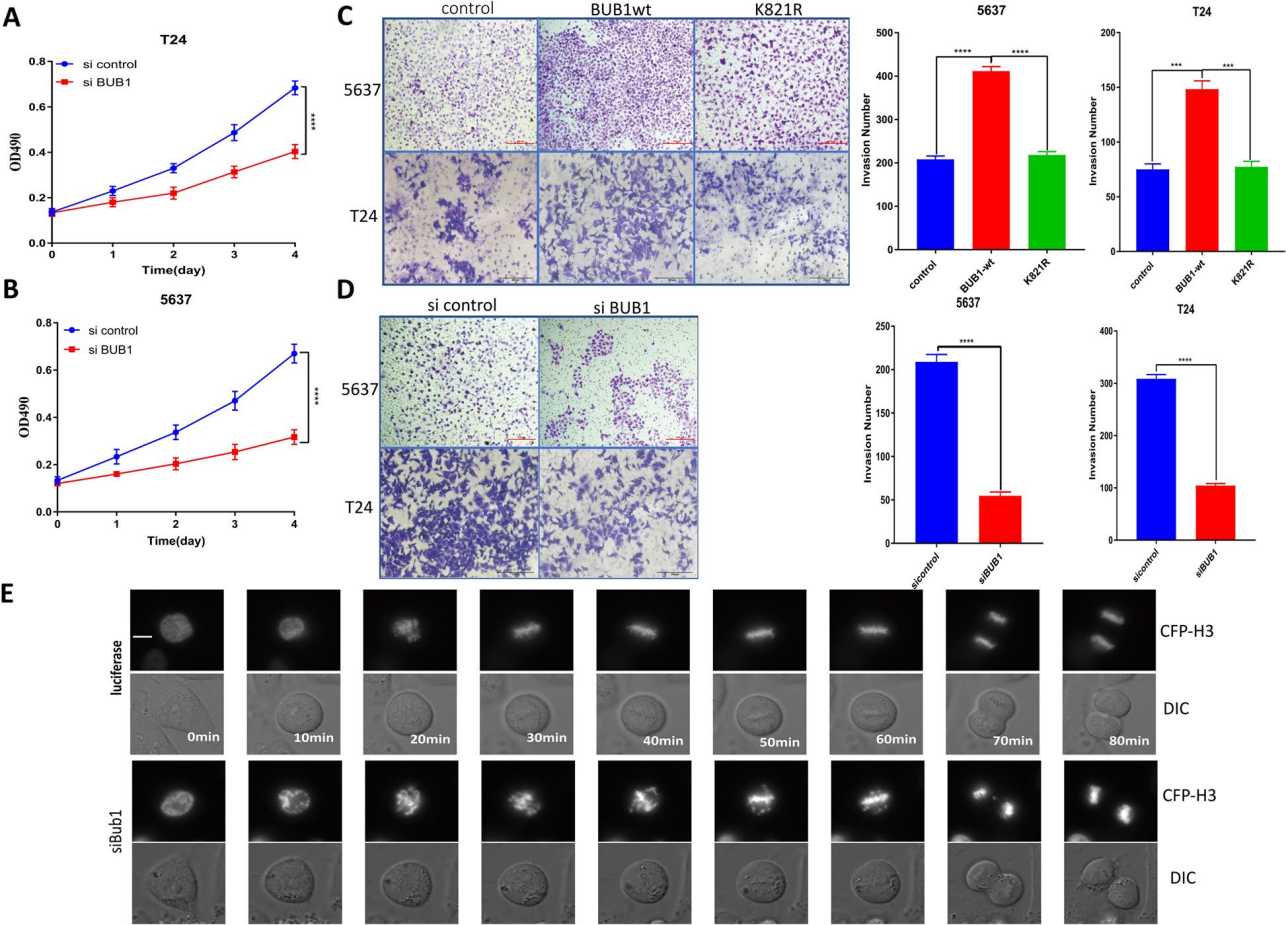
BUB1 regulated the proliferation and invasion of T24 and 5637 bladder cancer cells. **A**. T24 cells were transfected with sicontrol or siBUB1, and an MTT assay was used to evaluate cell proliferation. **B**. 5637 cell were transfected with sicontrol or siBUB1, and an MTT assay was used to evaluate cell proliferation. **C**. 5637 and T24 cells were transfected with control, BUB1 wild-type or BUB1 K821R vector, and Transwell assay was used to evaluate cell invasion ability. **D**. 5637 and T24 cells were transfected with si control or siBUB1, and Transwell assays were used to detect cell invasion ability. **E**. 5637 cell were cotransfected with BUB1 siRNA and the CFP-H3 plasmid for 48 h. Live-cell imaging was performed to monitor chromosome segregation during mitosis. The differential interference contrast (DIC) and CFP channels were recorded every 10 min for 16 h. Representative still images are shown. Scale bar 5 μm

The above results demonstrated the molecular functions of BUB1 in BCa, and we next analyzed the biological functions of BUB1 in tumor cells. The results of the MTT assay showed that transfection of specific siRNAs targeting BUB1 resulted in an effective reduction in cell proliferation (Fig. 6A, B). Overexpression of BUB1 via the wild-type vector significantly increased invasion, and downregulation of BUB1 in 5637 cells caused a significant reduction in their invasion (Fig. 6C, D). Interestingly, mutation of BUB1 (K821R) did not promote the invasion of 5637 cells (Fig. 6C). Downregulation of BUB1 in 5637 cells caused a significant reduction in their invasion, and this effect was also observed in T24 cells (Fig. 6D); similar results of BUB1 mutation (K821R) were also observed in T24 cells (Fig. 6C). In conclusion, BUB1 has an independent function in BCa cells that might contribute to the growth and spread of tumors. To understand the effects of BUB1 on BCa cell division, we used live-cell imaging with differential interference contrast microscopy to analyze the duration of mitosis in 5637 cells transfected with BUB1 targeting siRNAs. The results showed that BUB1 RNAi caused severe impairment of faithful chromosome segregation in 5637 cells (Fig. 6E). Numerous unaligned chromosomes and lagging chromosomes were observed during or after mitosis, an observation that can help to explain the observed effects on cell proliferation.

### Targeting the kinase activity of BUB1 suppressed BCa tumor growth in vivo

**Fig. 7:**
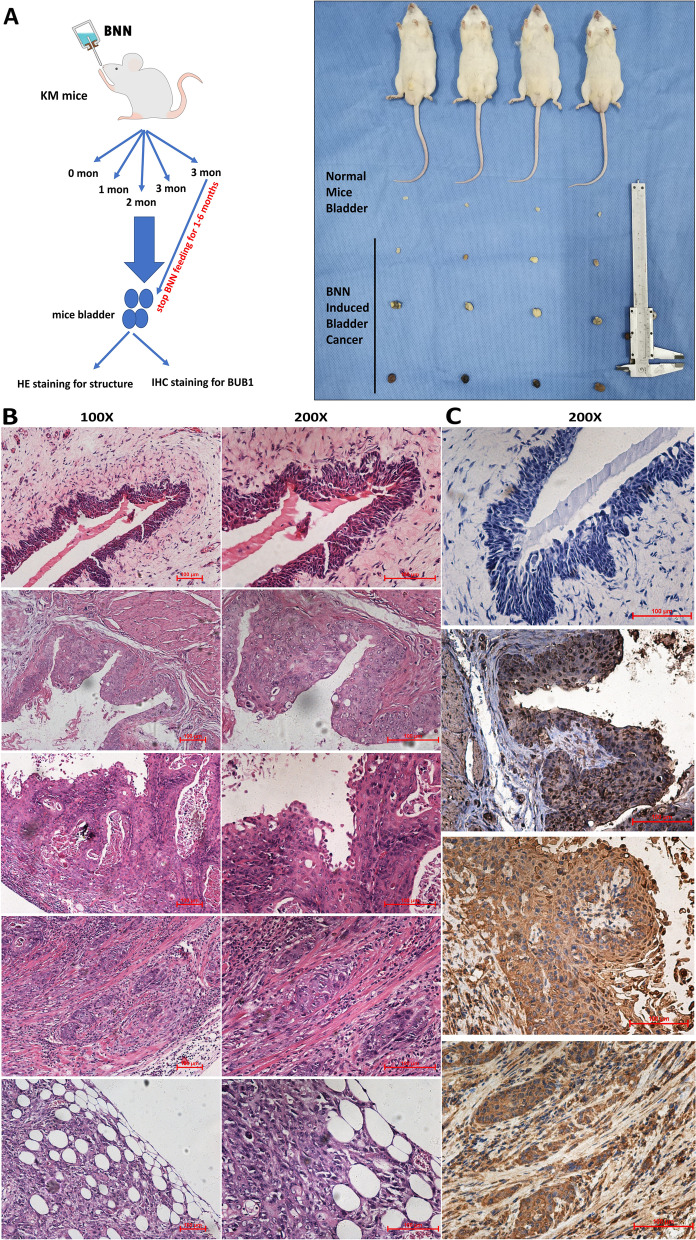
BUB1 expression was related to the occurrence and development of bladder cancer. **A**. Flow chart of the method used to establish the mouse model of spontaneous bladder cancer. **B**. H&E staining of the bladders of mice treated with 0.1% BNN in different stages of disease progression. The first row shows the normal structure of the mouse bladder epithelium; the second row shows the structure of mouse bladder cancer in situ; the third row shows the structure of mouse bladder papillary carcinoma; and the fourth and fifth rows show the structure of mouse invasive bladder cancer invading muscle tissue and adipose tissue. **C**. Immunohistochemical staining of the bladders of mice treated with 0.1% BNN in different stages of disease. The first row shows the expression of BUB1 in normal mouse bladder epithelium; the second row shows the expression of BUB1 in mouse bladder cancer in situ; the third row shows the expression of BUB1 in mouse bladder papillary carcinoma; and the fourth row shows the expression of BUB1 in mouse invasive bladder cancer

To further explore the relationship between BUB1 and the occurrence and development of bladder cancer in vivo, we established a model of spontaneous bladder cancer in KM mice via feeding water supplemented with 0.1% BNN (N-butyl-N-(4-hydroxybutyl) nitrosamine), which induces bladder cancer. We continued to feed mice 0.1% BNN for 3 months and killed three mice per month for 6 months after stopping BNN treatment. We excised the bladders and further embedded and sectioned the excised bladders (Fig. 7A). To explore the correlation between BUB1 expression and the occurrence and development of bladder cancer, we conducted immunohistochemical staining for BUB1 in these bladder sections. The results demonstrated that bladder cancer was successfully induced via feeding BNN-supplemented water and that BUB1 expression increased gradually with the progression of bladder cancer (Fig. 7B, C). The results in Fig. 7B show the complete evolution of bladder cancer progression based on H&E staining: from top to bottom, normal bladder epithelial structure, bladder cancer in situ, papillary bladder cancer and invasive bladder cancer. The immunohistochemical staining results for BUB1 further confirmed the role of BUB1 in the occurrence and development of bladder cancer. We found that BUB1 expression was higher in bladder cancer in situ than in normal bladder epithelium and that BUB1 expression in papillary bladder cancer and invasive bladder cancer was significantly elevated compared with that in bladder cancer in situ and normal bladder epithelium (Fig. 7C). The above results implied that BUB1 might be involved in the occurrence and progression of bladder cancer and that elevated BUB1 expression might drive the development of bladder cancer. The specific mechanism needs further research. These results did show that BUB1 expression was related to the occurrence and development of bladder cancer and that targeting BUB1 is a feasible potential treatment strategy.

**Fig. 8:**
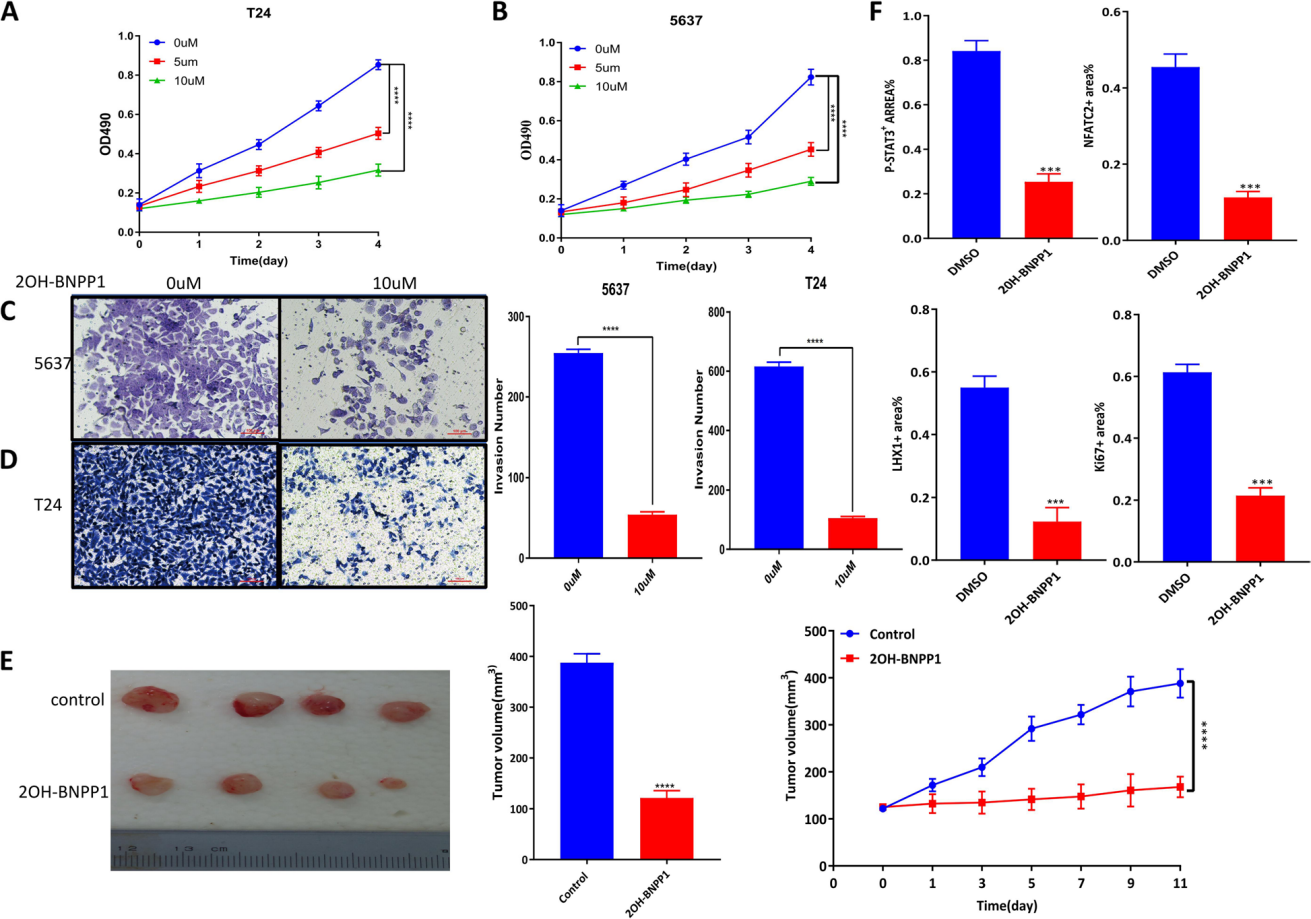
BUB1 Inhibitor 2OH-BNPP1 suppressed BCa Tumor Growth. **A** T24 cell were treated with 2OH-BNPP1 (5 or 10 μM) or vehicle (10% DMSO) for 96 h, and the number of viable cells was determined by an MTT assay. **B** 5637 cell were treated with 2OH-BNPP1 or vehicle (10% DMSO) for 96 h, and the number of viable cells was determined by an MTT assay. **C** 5637 cell were treated with 2OH-BNPP1 or vehicle (10% DMSO) for 24 h, and the number of invading cells was determined by an invasion assay. **D** T24 cell were treated with 2OH-BNPP1 or vehicle (10% DMSO) for 24 h, and the number of invading cells was determined by an invasion assay. **E** 5637 cell were implanted subcutaneously into male BALB/c mice. When the tumors became palpable, the mice were injected with either vehicle (10% DMSO in PBS) or 2OH-BNPP1 (100 mg/kg of body weight) for 10 days. Tumors were measured with calipers. **F** Quantitation of Ki-67, p-stat3, NFATC2 and LHX1 expression in 5637 xenograft tumors from each group. Specimens were obtained 10 days post treatment. IHC staining was scored according to the number of cells expressing the indicated proteins, and statistical analysis was performed (nonparametric Kruskal–Wallis test) to determine significance. See also Supplementary Fig. 3. **p < 0.01, *p < 0.05

To further investigate the antitumor activity of the BUB1 inhibitor, we first analyzed the ability of 2OH-BNPP1 to suppress the proliferation of BCa cell lines. T24 and 5637 cells were found to be sensitive to 2OH-BNPP1 treatment at 5 μm or 10 μm (Fig. 8A, B). We also found that 2OH-BNPP1 treatment at 10 μM inhibited the invasion of T24 and 5637 cells (Fig. 8C, D). To further investigate the effects of 2OH-BNPP1 on BCa xenograft tumor growth, 5637 cells were implanted subcutaneously into BALB/c mice. When the tumor volume was approximately 100 mm^3^, the mice were randomized and injected intratumorally with either DMSO or 2OH-BNPP1. Although robust subcutaneous tumors formed in DMSO-treated mice, tumor growth was observed to be reduced in 2OH-BNPP1-treated mice (Fig. 8E). As a result, we observed a reduced p-STAT3 level and decreased expression of the markers Ki67, NFATC2, and LHX1 in 2OH-BNPP1-treated tumors compared with DMSO-treated tumors by IHC analysis (Fig. 8F, Supplementary Fig. 3). Together, these results highlighted the potential of targeting BUB1 signaling to sensitize cells to BCa therapy.

## Discussion

BUB1 is upregulated in various types of human cancers, but the molecular function of BUB1 in promoting the occurrence and development of BCa has remained unknown. In the present study, we identified and provided evidence that BUB1 has oncogenic effects on bladder cancer transformation. We uncovered that BUB1 directly phosphorylated STAT3 at Ser727 and activated downstream genes to promote the development of BCa. The results showed that phosphorylation of serine 727 of STAT3 was required for the maximal transcriptional activation of STAT3, and the S727A mutant was confirmed to reduce transcriptional activation by 50% compared with that of wild-type STAT3 [29]. Furthermore, serine 727 phosphorylation enhanced STAT3 DNA binding and coactivator recruitment [30]. In this study, we reported a novel interaction between BUB1 and STAT3, which was first demonstrated by identification of the endogenous STAT3–BUB1 complex in BCa. Moreover, our data showed that BUB1 bound the STAT3/p300 complex and phosphorylated STAT3 at Ser727 (Figs. 1 and 2), suggesting that BUB1 is a positive regulator of STAT3 activity. Our data indicated that BUB1 not only mediated cancer cell mitosis but also contributed to transcriptional activation of STAT3 signaling in BCa cells. As supporting evidence, we also found that overexpression of BUB1 induced STAT3 phosphorylation and enhanced STAT3 activity and that knockdown of BUB1 with siRNA decreased STAT3 phosphorylation but not the total STAT3 level. Collectively, the above results suggested that BUB1 was essential for STAT3 phosphorylation. Moreover, the STAT3–BUB1 interaction led to an increase in endogenous STAT3 transcriptional activity in BCa cell lines, whereas the absence of BUB1 kinase activity led to significant impairment of STAT3 activity and the expression of the downstream genes NFATC2 and LHX1 (Fig. 3). LHX1 and NFATC2 have been identified as the transcription factors involved in epithelial progenitor cell development [31, 32]. These results suggested that BUB1 functioned a positive regulator of LHX1 and NFATC2 by increasing STAT3 activity to promote the proliferation of BCa cells (Fig. 4I). Surprisingly, we first found in our RNA-seq data that knockdown of BUB1 increased the mRNA expression levels of P53 and PTEN. Previous studies have indicated that P53 and PTEN are validated key tumor suppressors in regulating bladder cancer [33, 34]. Our studies supported a direct role for BUB1 in targeting BCa. However, the relationship among DNA replication, RNA transcription and spindle checkpoints in the cell cycle remains unclear. BUB1 is an essential protein of the spindle assembly checkpoint (SAC), which responds to improper attachment of kinetochores to microtubules [35, 36]. However, the role of BUB1 is not limited to the SAC in mitosis: Feng et al. reported that BUB1 can directly phosphorylate TRF1 and promote telomeric DNA replication [37]. Our work indicated that the SAC protein BUB1 is also critical for RNA transcription and that BUB1 might serve as a precise molecular switch by converting mitotic signaling to gene transcription signaling. Recent studies have discovered that BUB1 phosphorylates H2A-T120 and cdc20 at kinetochores during mitosis [4, 7, 8, 38]. Not only does H2A-pT120 recruit phosphorylated Sgo1 to kinetochores, H2A T120 phosphorylation affects H2A K119 ubiquitylation and H3K4 methylation to promote transcriptional activation [39, 40]. H2A T120 phosphorylation might influence the activity of HDAC1 and further influence transcription [41]. Cdc20, as a substrate of BUB1, is an activator of the anaphase-promoting complex or cyclosome (APC/C) [42]. APC/C tightly coordinates cell division and gene transcriptional network regulation [43]. Collectively, the above reports showed that BUB1 indirectly promotes transcription, while our studies identified the transcription factor STAT3 as a new BUB1 substrate and verified that BUB1 directly regulates transcription. In general, mitotic chromosome condensation is thought to silence transcription, which is reactivated at mitotic exit [44, 45]. However, a new study showed that mitotic cells maintained low levels of transcription and that the rate of transcription increased at mitotic exit [46]. In agreement with these findings, our research showed that another important spindle checkpoint protein, BUB1, is also involved in STAT3 transcriptional activation. We found that BUB1 phosphorylated STAT3 and further formed a complex, which bound to the promoter regions of NFATC2 and LHX1 and activated their transcription. Based on the above results, we speculated that in addition to Sgo1 and Pol II, BUB1 directly recruits and activates STAT3 transcription in dense mitotic chromatin. Our results were consistent with the idea of a noncanonical function of transcription allowing chromosomal regulators to be embedded in dense mitotic chromatin. How BUB1 switches from regulating the mitotic spindle checkpoint to RNA transcription is an exciting question for future research. The findings from this research would increase our understanding of the crosstalk between RNA transcription and the mitotic spindle checkpoint. Our results, together with recent reports, further develop the concept of mitotic control of cancer cell transcription.

## Conclusion

In this study, we demonstrated the role of BUB1 as a novel, positive regulator of STAT3 activity. BUB1 interacted with STAT3 and phosphorylated STAT3 at Ser727. Furthermore, BUB1 recruited STAT3 and the transcriptional coactivator p300 to form a complex, and these events collectively facilitated the enhancement of serine phosphorylation-dependent STAT3 transcriptional activity. Finally, BUB1 also enhanced NFATC2- and LHX1-induced BCa cell proliferation mediated by STAT3. A pharmacological inhibitor of BUB1 (2OH-BNPP1) inhibited the growth of BCa cell xenografts. The BUB1 kinase may be an attractive candidate for therapeutic targeting in BCa.

## Supplementary Information

**Additional file 1: Supplementary Figure 1.** Supplementary paired bladder cancer tissue western blot. The protein expression of BUB1 in bladder cancer tissue is higher compared with the normal bladder tissue.

**Additional file 2: Supplementary Figure 2.** BUB1 regulates the signaling of its target gene STAT3. A: KEGG annotation showing the involvement of the identified siBUB1 and STAT3-cotargeted genes in the JAK-STAT3 signaling pathway. B: KEGG annotation showing the involvement of the identified siBUB1 and STAT3-cotargeted genes in the WNT signaling pathway. C: KEGG annotation showing the involvement of the identified siBUB1 and STAT3-cotargeted genes in the P53 signaling pathway. D: KEGG annotation showing the involvement of the identified involved siBUB1 and STAT3-cotargeted genes in the PTEN signaling pathway. E: STAT3 binding site in the promoter of the LHX1 gene. F: STAT3 binding site in the promoter of the NFATC2 gene.

**Additional file 3: Supplementary Figure 3.** Assessment of P-STAT3, LHX1, NFATC2 and Ki67 protein expression by immunohistochemical staining in 5637 cell xenografts after treatment with the inhibitor-2OH-BNPP1, related to Fig. 8. Representative images of P-STAT3, LHX1, NFATC2 and Ki67 protein expression in 5637 cell xenograft tumors after2OH-BNPP1 treatment; specimens were obtained 10 days post treatment.

**Additional file 4: Table S1.** Patient Demographics

**Additional file 5. Table S2.** Primers sequences and human NFATC2 and LHX1 promoters.

**Additional file 6: Table S3.** A summary of differentially expressed gene lists in 5637 cell transfected with si control and si BUB1. 

**Additional file 7: Supplementary Table S4.** The target genes were co-targeted by both BUB1 and STAT3. 

## Data Availability

All data generated or analyzed during this study are included either in this article or in the supplementary information files.

## Abbreviations

BCa: Bladder cancer
UC: Urothelial carcinoma
BUB1: Budding uninhibited by benzamidazole 1
PI3K: Phosphoinositide 3-kinase
TCGA: The Cancer Genome Atlas
MAPKs: Mitogen-activated protein kinases
KT-MT: Kinetochore-microtubule
SAC: Spindle assembly checkpoint
APC/C: Anaphase Promoting Complex/Cyclosome
