# Supplementary figures and images for: BUB1 drives the occurrence and development of bladder cancer by mediating the STAT3 signaling pathway
Source: J Exp Clin Cancer Res. 2021 Dec 1;40:378. doi: 10.1186/s13046-021-02179-z (PMC8638147; doi:10.1186/s13046-021-02179-z)

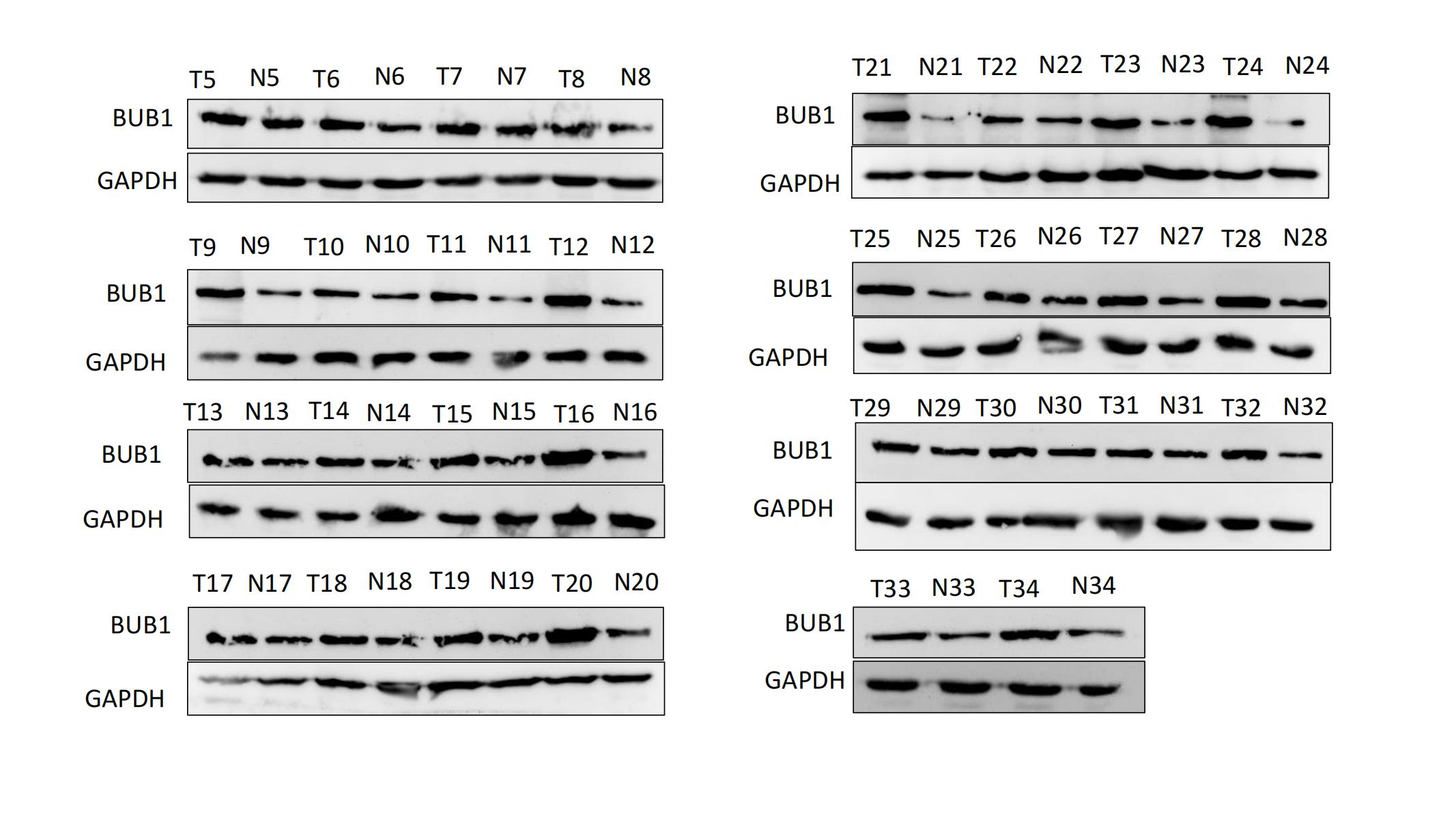

Supplement: Supplementary file 1 — Additional file 1: Supplementary Figure 1. Supplementary paired bladder cancer tissue western blot. The protein expression of BUB1 in bladder cancer tissue is higher compared with the normal bladder tissue. [file 13046_2021_2179_MOESM1_ESM.jpg]

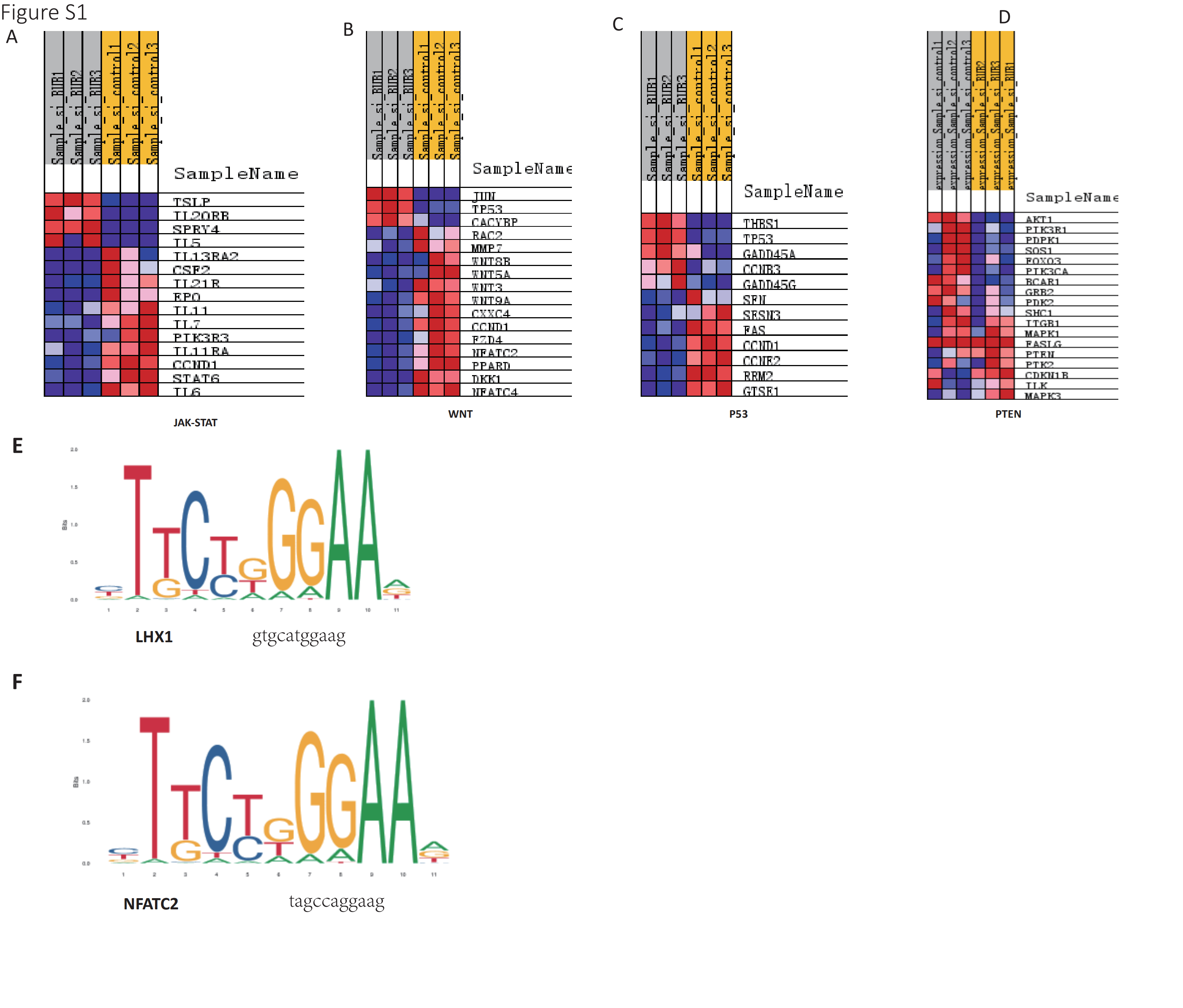

Supplement: Supplementary file 2 — Additional file 2: Supplementary Figure 2. BUB1 regulates the signaling of its target gene STAT3. A: KEGG annotation showing the involvement of the identified siBUB1 and STAT3-cotargeted genes in the JAK-STAT3 signaling pathway. B: KEGG annotation showing the involvement of the identified siBUB1 and STAT3-cotargeted genes in the WNT signaling pathway. C: KEGG annotation showing the involvement of the identified siBUB1 and STAT3-cotargeted genes in the P53 signaling pathway. D: KEGG annotation showing the involvement of the identified involved siBUB1 and STAT3-cotargeted genes in the PTEN signaling pathway. E: STAT3 binding site in the promoter of the LHX1 gene. F: STAT3 binding site in the promoter of the NFATC2 gene. [file 13046_2021_2179_MOESM2_ESM.png]

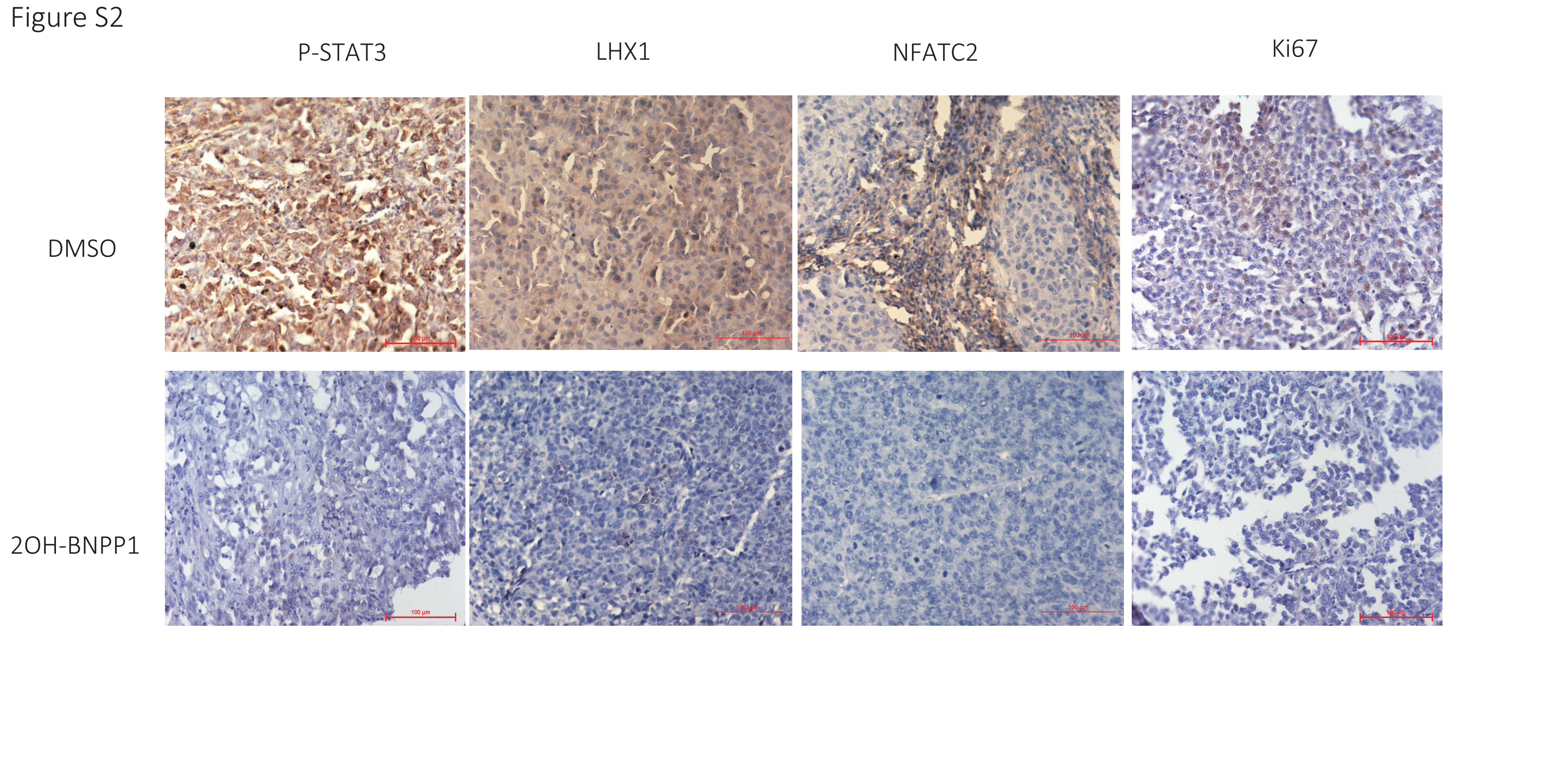

Supplement: Supplementary file 3 — Additional file 3: Supplementary Figure 3. Assessment of P-STAT3, LHX1, NFATC2 and Ki67 protein expression by immunohistochemical staining in 5637 cell xenografts after treatment with the inhibitor-2OH-BNPP1, related to Fig. 8. Representative images of P-STAT3, LHX1, NFATC2 and Ki67 protein expression in 5637 cell xenograft tumors after2OH-BNPP1 treatment; specimens were obtained 10 days post treatment. [file 13046_2021_2179_MOESM3_ESM.png]
