# Supplementary material for: BUB1 drives the occurrence and development of bladder cancer by mediating the STAT3 signaling pathway
Source: J Exp Clin Cancer Res. 2021 Dec 1;40:378. doi: 10.1186/s13046-021-02179-z (PMC8638147; doi:10.1186/s13046-021-02179-z)
Supplement: Supplementary file 4 — Additional file 4: Table S1. Patient Demographics [file 13046_2021_2179_MOESM4_ESM.docx]

| Cystectomy Patients,n |  |  | 34 |  |
| --- | --- | --- | --- | --- |
| Age at Cystectomy |  |  |  |  |
| Mean(SD) |  |  | 62.73(6.11) |  |
| Gender |  |  |  |  |
| Male,n(%) |  |  | 21(62%) |  |
| Female,n(%) |  |  | 13(38%) |  |
| T stage |  |  |  |  |
| Tis,n(%) |  |  | 1(3%) |  |
| Ta,n(%) |  |  | 2(6%) |  |
| T1,n(%) |  |  | 2(6%) |  |
| T2,n(%) |  |  | 10(29%) |  |
| T3,n(%) |  |  | 16(47%) |  |
| T4,n(%) |  |  | 3(9%) |  |
| Grade |  |  |  |  |
| 1,n(%) |  |  | 2(6%) |  |
| 2,n(%) |  |  | 11(32%) |  |
| 3,n(%) |  |  | 20(59%) |  |
| Cis alone,n(%) |  |  | 1(3%) |  |
| N stage |  |  |  |  |
| Lymph Node Positive,n(%) |  |  | 3(9%) |  |
| Lymphovascular Invasion,n(%) |  |  | 5(15%) |  |
| Death,n(%) |  |  | 9(26%) |  |

Table S1

Patient Demographics
